# Immune analysis of expression of IL-17 relative ligands and their receptors in bladder cancer: comparison with polyp and cystitis

**DOI:** 10.1186/s12865-016-0174-8

**Published:** 2016-10-03

**Authors:** Yanbo Liu, Wanguo Yang, Lijing Zhao, Zuowen Liang, Weigao Shen, Qinlong Hou, Zhenjiang Wang, Jing Jiang, Sun Ying

**Affiliations:** 1 The Clinical Immunology Research Centre, Beihua University, No. 3999 Binjiang Road, Jilin City, Jilin China; 2 The Pathophysiology department of basic medical college, Jilin University, NO. 126, Xinmin street, Changchun City, Jilin China; 3 The Andrology Department of the First Hospital, Jilin University, NO. 71 Xinmin street, Changchun City, Jilin China; 4 Guy’s Hospital, AALB, 5th Floor Tower Wing, London, SE1 9RT UK

**Keywords:** IL-17 relative ligands, IL-17 relative receptors, Bladder cancer, Bladder polyp, Cystitis

## Abstract

**Background:**

Bladder cancer, cystitis and bladder polyp are the most common urinary system diseases all over the world. Our former research results show that IL-17A and IL-17 F contribute to the pathogenesis of benign prostatic hyperplasia (BPH) and prostate cancer (Pca) while IL-17E interacting with IL-17RB might have an anti-tumor effect.

**Results:**

Using imunohistochemistry, we systemically compared immunoreactivity of ligands (IL-17A, E and F) and receptors (IL-17RA, IL-17RB and IL-17RC) of IL-17 family, infiltration of inflammatory cells and changes of structural cells (fibroblast cells, smooth muscle and vascular endothelial cells) in sections of bladder tissues from subjects with bladder cancer, cystitis and bladder polyp. Compared with subjects with cystitis, immunoreactivity for IL-17A, IL-17 F and IL-17RC was significantly elevated in the group of bladder cancer (*p* < 0.01), while immunoreactivity of IL-17E, IL-17RA and IL-17RB, and the infiltrating neutrophils were decreased (*p* < 0.05). The numbers of infiltrating lymphocytes and phagocytes and CD31^+^ blood vessels and immunoreactivity of CD90^+^ fibroblasts were also elevated in patients with bladder cancer compared with those of cystitis. The patterns of IL-17 ligands and receptors, and inflammatory cells and structural cells varied in cystitis, bladder polyp and bladder cancer. In bladder cancer, immunoreactivity of IL-17E and IL-17 F was positively correlated with smooth muscles and lymphocytes, respectively. In addition, immunoreactivity of IL-17A and IL-17E was positively correlated with their receptors IL-17RA and IL-17RB respectively.

**Conclusions:**

The data suggest that changed patterns of expression of the IL-17 cytokine family ligands and receptors might be associated with infiltration of inflammatory cells and structural cells (CD90^+^ fibroblasts and CD31^+^ blood vessels), which might also contribute to occurrence and development in bladder cancer.

**Electronic supplementary material:**

The online version of this article (doi:10.1186/s12865-016-0174-8) contains supplementary material, which is available to authorized users.

## Background

There are about 400,000 individuals with bladder cancer in the world and 150,000 patients die from the disease every year [1, 2]. In the United States bladder cancer is the 5th most common type of cancer with 72,500 new cases and 15, 200 deaths occurring in 2013 [3], while cystitis and polyp are considered as high-risk for bladder cancer [4]. It has been shown that multiple bladder polyps and cystitis are easy to develop malignant disease depending on the scope and duration of those relative diseases [5]. Although many factors may be associated with the pathogenesis and mechanisms of above diseases, some cytokines, including tumor necrosis factor-α (TNF-α), interleukin-17 (IL-17) cytokine family and interferon (IFN) are considerably involved in the occurrence and development of cystitis, bladder polyp and bladder cancer [6, 7].

The IL-17 cytokine family includes six ligands (IL-17A to IL-17 F) and five receptors (IL-17RA to IL-17RE). Because of their unique and distinct biological functions, most studies are focused on IL-17A and IL-17E in tumors [8, 9]. In addition, IL-17 F has also been studied because of its high molecular homology and similar biological functions with IL-17A [10]. Previous studies have shown that both IL-17A and IL-17E can bind to their receptors IL-17RA and IL-17RB, while IL-17 F can bind to its own receptor IL-17RC and/or IL-17RA to fulfill their biological function. It has been indicated that IL-17A and IL-17 F are key pro-inflammatory cytokines in the pathogenesis of inflammatory and autoimmune diseases [11]. Compared with IL-17A/IL-17 F, IL-17E plays an important role in the pathogenesis of asthma and atopic diseases through binding to the heterodimeric complex of IL-17RA/IL-17RB [12]. On the other hand, some studies have also indicated the paradoxes of the pro-tumor or anti-tumor activity of IL-17 family relative ligands [11, 13]. Previous data have shown that macrophages secreting IL-17 family cytokines may play important roles in the pathogenesis of malignant tumors [14]. For example, it has been shown that IL-17A transcripts in peripheral blood mononuclear cells [15] and serum concentrations of IL-17A [16] were significantly higher in peripheral blood mononuclear cells in subjects with bladder cancer than those of controls. In animal experiments, it has been reported that IL-17A promoted bladder cancer growth [17], while IL-17-producing γδ T cells possibly play a key role in the Bacillus Calmette-Guéri (BCG)-induced recruitment of neutrophils to the bladder, which is essential for the antitumor activity against bladder cancer [18]. However, the expression and location of other IL-17 family cytokines and their receptors, and their relationships to bladder relative disease progression, inflammatory cellular infiltration and structural changes are still largely unclear in cystitis, bladder polyp and bladder cancer.

In this study we expanded our previous observations in prostate cancer [19] and hypothesized that in bladder tissues, both infiltrating inflammatory cells and structural cells can express IL-17 family cytokines and relevant receptors, and that such expressions can affect not only tissue remodelling but also angiogenesis, which are associated with disease severity and tumorigenesis. We compared expression and location of IL-17 cytokine family IL-17A, IL-17E and IL-17 F and their receptors IL-17RA, IL-17RB and IL-17RC in tissues derived from subjects with cystitis, bladder polyp and bladder cancer in parallel. We also analyzed the relationships between expression of these IL-17 family cytokines and their receptors, infiltration of inflammatory cells and changes of structural cells in these diseases.

## Methods

### Patients and specimens

The study was approved by the Hospital Ethics Committees of Urinary System Diseases Prevention and Treatment Research Centre of the Affiliated Hospital of Beihua University, Jilin City, Jilin Province, People’s Republic of China (approval reference: 2012BH006), as formulated in the World Medical Association Declaration of Helsinki. Written and informed consents were provided by all subjects participated, including 23 patients who were biopsied and diagnosed as cystitis and 6 patients with hyperplastic bladder polyps from January 2012 to December 2014. Tumor tissues were collected from 80 patients with transitional cell carcinoma during surgical resection during the same time. The above diseases were diagnosed as previously described [20, 21]. The clinical characteristics of subjects involved in this study are summarized in Table 1.

**Table 1:** Clinical characteristics of the subjects in this study

Status	Age (median)	Sources	Pathological characteristics
Cystitis *n* = 23	63 (22–83) Male 18 Female 5	Endoscopic biopsies	
Polyp *n* = 6	60 (35–84) Male 5 Female 1	Endoscopic biopsies	Hyperplastic polyp
Bladder cancer *n* = 80	64 (45–84) Male 66 Female 14	Endoscopic biopsies or resection specimens	Transitional cell carcinoma

### Immunohistochemistry

Immunohistochemistry was applied in paraffin sections (4 μm thickness) to evaluate expression and location for IL-17 family relative ligands (IL-17A, IL-17E and IL-17 F) and their relative receptors (IL-17RA, IL-17RB and IL-17RC), infiltration of inflammatory cells (T lymphocytes, macrophages, mast cells and neutrophils) and relevant structural cells (CD90^+^ fibroblast cells, smooth muscle and CD31^+^ vascular endothelial cells) as described previously [19]. The sources of antibodies and their optimal dilutions are indicated as Additional file 1: Table S1. DAB kit (diaminobenzidine, ZhongShan Golden Bridge Biological Company, Beijing, China) was used to evaluate positive signals of immunestaining while applying an irrelevant, matching isotype of IgG as substitution of the primary antibody was used as negative control. All slides were blindly analysed by two observers using an Olympus microscope connected with a computer running Image Pro Plus 6.0 software (Media Cybernetics, Maryland, USA). Global immunoreactivity of IL-17 family relative cytokines as well as CD90^+^ cells was quantified as the percentage staining of the total stainable area of the sections defined with the haematoxylin counterstaining, while inflammatory cells (such as lymphocytes, macrophages, neutrophils and mast cells) and CD31^+^ blood vessels were quantified as the numbers of immunoreactive cells per unit area of the entire sections [18].

### Statistical analysis

All the labeling area stained by the DAB in the immunohistochemistry procedure was measured, which based on the principle of RGB color deconvolution using software of NIH-Image J plugin. According to the sizes of slice sides 6–20 fields, at 200 × original magnification, were analyzed, and data were digitized and transferred to NIH-Image J software. Data were analyzed with a statistical package (Minitab for Windows, Minitab Release 9.2; Minitab, Inc, State College, PA). Nonparametric Mann-Whitney test was used to analyze difference between groups. For all tests, *p* < 0.05 were considered as significant. Data are presented in the Figures as the median ± SD.

## Results

### Immunoreactivity and location of IL-17A and IL-17RA

**Fig. 1:**
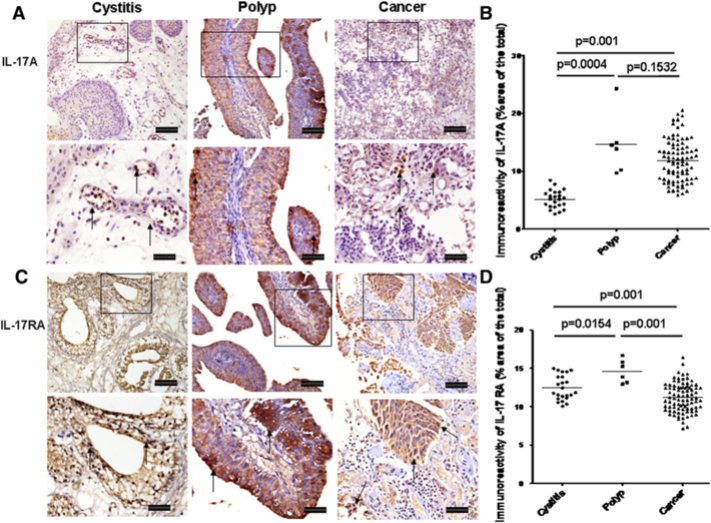
Immunoreactivity of IL-17A and IL-17RA in cystitis, polyp and bladder cancer tissues. **a** Representative photomicrographs of IL-17A expression in bladder sections from subjects with cystitis (*n* = 23), polyp (*n* = 6) and bladder cancer (*n* = 80) (original magnification × 10 and 20). **b** Quantitative analysis of IL-17A expression area of bladder sections (% of whole sections). **c** Representative photomicrographs of IL-17RA expression in bladder sections from cystitis, polyp and cancer. **d** Quantitative analysis of IL-17RA expression area of bladder sections (% of whole sections). Data are expressed as the mean ± SEM. Arrows show examples of positively stained cells. The scale bar means 100 μm

Immunohistochemical staining analysis showed that global IL-17A expression was significantly elevated in tissue sections from bladder polyp and bladder cancer compared with cystitis (Fig. 1a and b, *p* = 0.001 and *p* = 0.001, respectively), while there was no significant difference in global IL-17A immunoreactivity between bladder polyp and bladder cancer (*p* = 0.170). IL-17A expression located mainly in mononuclear cells, transitional epithelial cells, malignant cells and vascular endothelial cells in bladder cancer (Fig. 1a). In contrast to IL-17A, global IL-17RA immunoreactivity was significantly elevated in cystitis and polyp compared with those of bladder cancer (Fig. 1c and d, *p* = 0.001 and *p* = 0.001, respectively), while global IL-17RA immunoreactivity was significantly higher in polyps than those of cystitis (*p* = 0.015). The location of IL-17RA immunoreactivity was similar to IL-17A, which mainly located in transitional epithelial cells, mononuclear cells in interstitium and cancerous cells (Fig. 1c).

### Immunoreactivity and location of IL-17E and IL-17RB

**Fig. 2:**
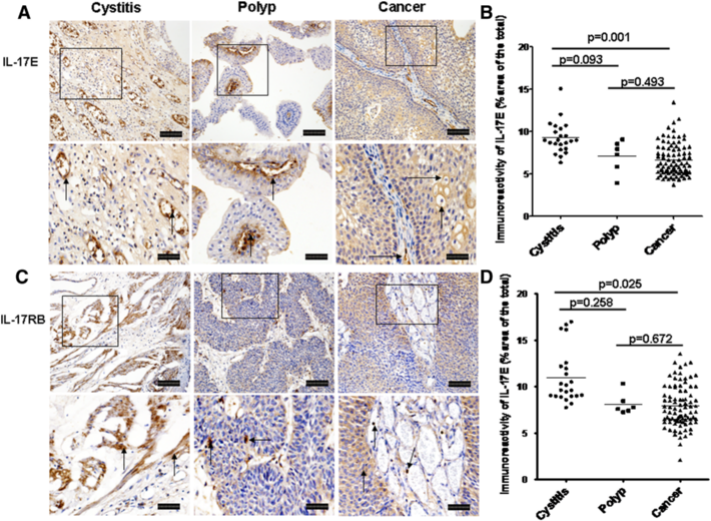
Immunoreactivity of IL-17E and IL-17RB in cystitis, polyp and bladder cancer tissues. **a** Representative photomicrographs of IL-17E expression in bladder sections from subjects with cystitis (*n* = 23), polyp (*n* = 6) and bladder cancer (*n* = 80) (original magnification × 10 and 20). **b** Quantitative analysis of IL-17E expression area of bladder sections (% of whole sections). **c** Representative photomicrographs of IL-17RB expression in bladder sections from cystitis, polyp and cancer. **d** Quantitative analysis of IL-17RB expression area of bladder sections (% of whole sections). Data are expressed as the mean ± SEM. Arrows show examples of positively stained cells. The scale bar means 100 μm

Global IL-17E expression was significantly elevated in cystitis compared with those of bladder cancer (Fig. 2a and b, *p* = 0.001), while there was no significant difference between cystitis and polyp (Fig. 2a and b, *p* = 0.093). Similar to IL-17A, IL-17E expression was predominantly observed in mononuclear cells, transitional epithelial cells and vascular endothelial cells (Fig. 2a). Global IL-17RB immunoreactivity was significantly higher in the tissue sections from cystitis than that from bladder cancer (Fig. 2c and d, *p* = 0.025). IL-17RB mainly located in mononuclear cells, vascular endothelial cells, smooth muscle cells and some cancerous cells (Fig. 2c).

### Immunoreactivity and location of IL-17 F and IL-17RC

**Fig. 3:**
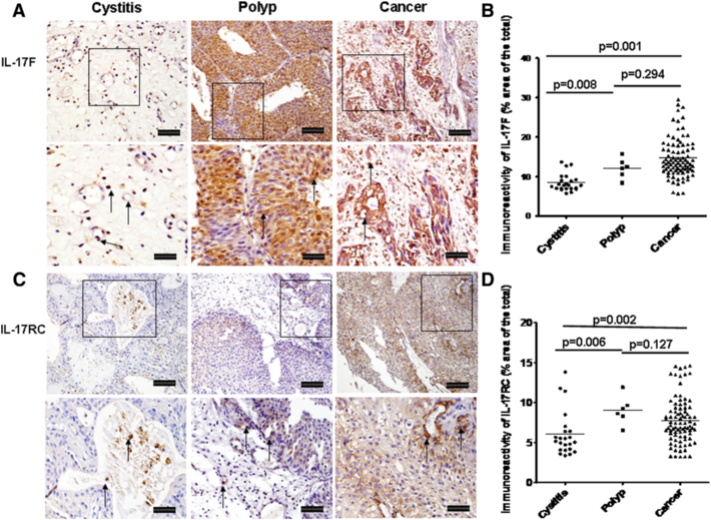
Immunoreactivity of IL-17 F and IL-17RC in cystitis, polyp and bladder cancer tissues **a** Representative photomicrographs of IL-17 F expression in bladder sections from subjects with cystitis (*n* = 23), polyp (*n* = 6) and bladder cancer (*n* = 80) (original magnification × 10 and 20). **b** Quantitative analysis of IL-17 F expression area of bladder sections (% of whole sections). **c** Representative photomicrographs of IL-17RC expression in bladder sections from cystitis, polyp and cancer. **d** Quantitative analysis of IL-17RC expression area of bladder sections (% of whole sections). Data are expressed as the mean ± SEM. Arrows show examples of positively stained cells. The scale bar means 100 μm

Global expression for IL-17 F was significantly greater in the tissue sections from bladder cancer and polyp compared with cystitis (Fig. 3a and b, *p* = 0.001and *p* = 0.008, respectively), but there was no significant difference between bladder cancer and polyp (Fig. 3a and b, *p* = 0.294). IL-17 F mainly expressed in mononuclear cells, transitional epithelial cells, vascular endothelial cells and some malignant cells (Fig. 3a). Because IL-17 F can bind not only to IL-17RA but also to IL-17RC to exert biological effects, we also evaluated global immunoreactivity for IL-17RC in all the sections. Global expression for IL-17RC was significantly increased in polyp and bladder cancer compared with cystitis (Fig. 3c and d, *p* = 0.007, *p* = 0.002), while there was no significant difference between polyp and bladder cancer (*p* = 0.127). Similar to IL-17 F, IL-17RC immunoreactivity located principally in mononuclear cells, transitional epithelial cells, vascular endothelial cells and some malignant cells in bladder cancer (Fig. 3c).

### Infiltration of T lymphocytes and macrophages

**Fig. 4:**
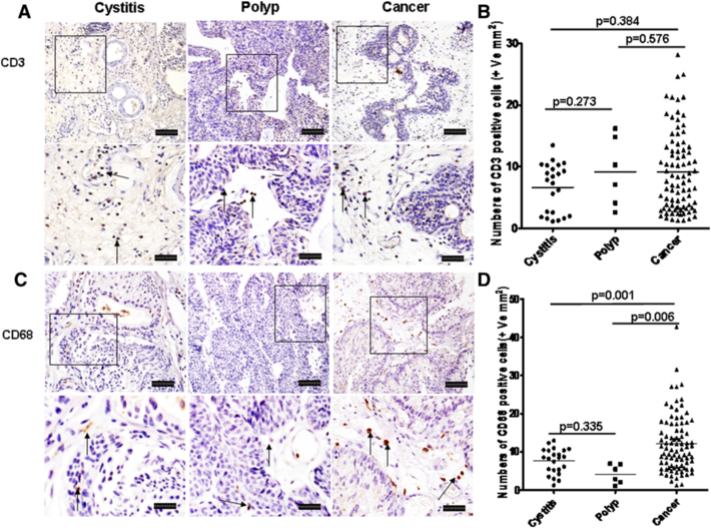
Infiltration T cells and macrophages in cystitis, polyp and bladder cancer tissues. **a** and **c** Representative photomicrographs of CD3^+^ T cells and CD68^+^ macrophages in bladder sections from subjects with cystitis (*n* = 23), polyp (*n* = 6) and bladder cancer (*n* = 80) (original magnification × 10 and 20). **b** and **d** Quantitative analysis of numbers of CD3^+^ T cells and CD68^+^ macrophages per unit area of bladder sections. Data are expressed as the mean ± SEM. Arrows show examples of positively stained cells. The scale bar means 100 μm

Compared with cystitis, the numbers of CD3^+^ lymphocytes increased in both polyps and bladder cancers but did not achieve statistically significance (Fig. 4a and b, *p* = 0.273, *p* = 0.384 respectively). These cells located predominately in the stroma of polyp and bladder cancer. Like CD3^+^ lymphocytes, CD68^+^ macrophages also located mainly in stroma of the bladder tissues. The median number of CD68^+^ macrophages was significantly elevated in bladder cancers compared with cystitis and polyps (Fig. 4c and d, *p* = 0.001, *p* = 0.006 respectively). However, there was no obvious difference between cystitis and polyp (*p* = 0.3774).

### Infiltration of neutrophils and mast cells

**Fig. 5:**
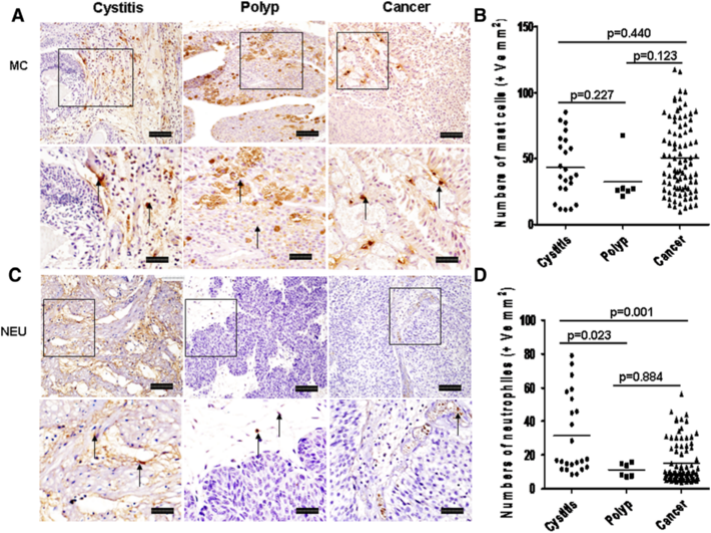
Infiltration of mast cellsand neutrophils in cystitis, polyp and bladder cancer tissues. **a** and **c** Representative photomicrographs of tryptase^+^ mast cells and elastase^+^ neutrophils in bladder sections from subjects with cystitis (*n* = 23), polyp (*n* = 6) and bladder cancer (*n* = 80) (original magnification × 10 and 20). **b** and **d** Quantitative analysis of numbers of tryptase^+^ mast cells and elastase^+^ neutrophils per unit area of bladder sections. Data are expressed as the mean ± SEM. Arrows show examples of positively stained cells. The scale bar means 100 μm

In order to explore other inflammatory cells infiltration, we further detected elastase^+^ neutrophils and tryptase^+^ mast cells in the sections from bladder tissues. Both mast cells and neutrophils mainly located in stroma. The median numbers of mast cells were slightly higher in cystitis and bladder cancer, but no statistical difference compared with polyps (Fig. 5a and b, *p* = 0.227, *p* = 0.123, respectively), and again there was no significant difference was achieved between cystitis and bladder cancer (*p* = 0.440) (Fig. 5a and b). The median number of neutrophils in cystitis compared was significantly elevated with polyp and bladder cancer (Fig. 5c and d, *p* = 0.023, *p* = 0.001, respectively), while there was no obvious difference between polyp and bladder cancer (*p* = 0.884) (Fig. 5c and d).

#### Changing of structural cells

**Fig. 6:**
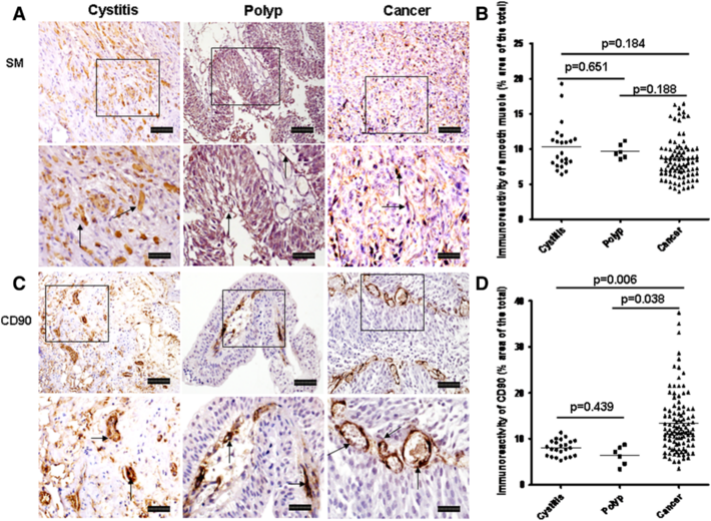
Expression for smooth muscle and CD90^+^ fibroblasts in cystitis, polyp and bladder cancer tissues. **a** and **c** Representative photomicrographs of smooth muscle and CD90^+^ fibroblasts with cystitis (*n* = 23), polyp (*n* = 6) and bladder cancer (*n* = 80) (original magnification × 10 and 20). **b** and **d** Quantitative analysis of smooth muscle and CD90^+^ fibroblast immunoreactive area of bladder sections (% of whole sections). Data are expressed as the mean ± SEM. Arrows show examples of positively stained cells. The scale bar means 100 μm

**Fig. 7:**
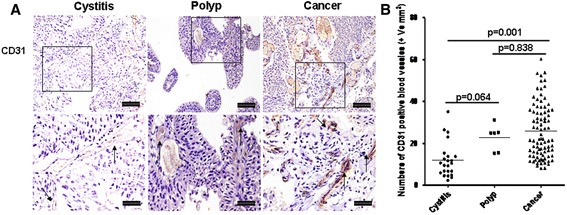
CD31^+^ blood vessels in cystitis, polyp and bladder cancer tissues. **a** Representative photomicrographs of CD31^+^ blood vessels with cystitis (*n* = 23), polyp (*n* = 6) and bladder cancer (*n* = 80) (original magnification × 10 and 20). **b** Quantitative analysis of numbers of CD31^+^ blood vessels per unit area of bladder sections. Data are expressed as the mean ± SEM. Arrows show examples of positively stained endothelial cells. The scale bar means 100 μm

We also evaluated the changing of some types of structural cells in tissues such as α-actin^+^ smooth muscle, CD90^+^ fibroblast cells and CD31^+^ vascular endothelial cells. The median expression of smooth muscle cells was slightly decreased in bladder cancer, but there were no statistical differences among three groups (Fig. 6a and b). The median immunoreactivity of CD90^+^ cells per unit area of the tissue was dramatically elevated in bladder cancer compared with cystitis and polyp (Fig. 6c and d, *p* = 0.006, *p* = 0.038, respectively), but no statistical difference was observed between cystitis and polyps (Fig. 6c and d, *p* = 0.439). The median number of CD31^+^ blood vessels was significantly elevated in the tissue sections from bladder cancer compared with those of cystitis (Fig. 7a and b, *p* = 0.001). Although the median number of CD31^+^ blood vessels also increased in polyps, this did not achieve statistical significance compared with cystitis (Fig. 7a and b, *p* = 0.064).

### The relationship of expression for IL-17 cytokine family ligands and their receptors, infiltration of inflammatory cells and changes in structural cells in bladder cancer

**Fig. 8:**
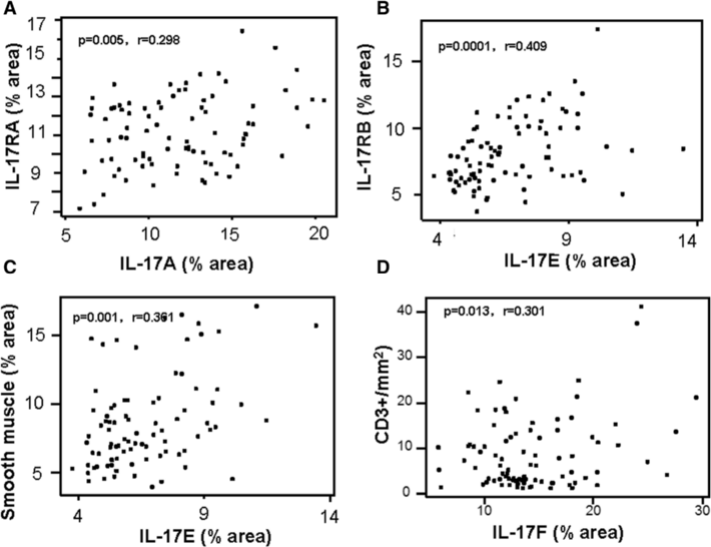
The relationship between IL-17 family related ligands, receptors, inflammatory cells and structural cells in bladder cancer. **a** Positive correlation between IL-17A and IL-17RA. **b** Positive correlation between IL-17E and IL-17RB. **c** Positive correlation between smooth muscle and IL-17E. **d** Positive correlation between IL-17 F expression and infiltration of lymphocytes

In order to explore the correlations between IL-17 family cytokines, and inflammatory cells as well as structural cells, we further analyzed correlations of global expression for IL-17 family ligands and their receptors and relevant phenotypes of cells in bladder cancer. Of great interest, global immunoreactivity for IL-17RA significantly correlated with its ligand IL-17A (Fig. 8a, r = 0.298, *p* = 0.005), while IL-17E significantly correlated with its receptor IL-17RB (Fig. 8b, r = 0.409, *p* = 0.0001). In addition, global immunoreactivity for α-actin^+^smooth muscle was significantly correlated with IL-17E (Fig. 8c, r = 0.301, *p* = 0.001), while the numbers of CD31^+^ blood vessels significantly correlated with IL-17 F in bladder cancer tissues (Fig. 8d, r = 0.301, *p* = 0.013).

## Discussion

Although a numbers of previous studies seem to prove that IL-17 family cytokines have the capability to promote occurrence and development of cystitis, polyp and bladder cancer [22–25], there is a lack of systematically comparison study of IL-17 family ligands and their corresponding receptors in these urinary system diseases. Here we analyzed the IL-17 family cytokines IL-17A, E and F and their receptors IL-17RA, RB and RC in bladder tissues from patients with cystitis, polyp and bladder cancer. We also simultaneously examined inflammatory cellular infiltration, structural changes and angiogenesis in these urinary disorders.

Accumulation of infiltrating inflammatory cells is a typical feature of cystitis, possibly in bladder cancer as well. However, proinflammatory responses are double- edged sword with protective and tumorgenesis roles. On the one hand, such accumulated phagocytes might cause further inflammation in bladder tissues, which possibly exerts an anti-tumor activity, through generating more inflammatory mediators, including IL-17 family cytokines. On the other hand, these mediators and cytokines, in turn, might promote and enhance the abnormal cellular proliferation associated with polyp and bladder cancer, through generating various mediators, including inflammatory cytokines such as interleukin-6 (IL-6) and cellular growth factor (TGF-β) [23, 25, 26]. Our data showed that expression of IL-17 cytokine family ligands and their relevant receptors was accompanied with markedly distinct profiles of infiltration of inflammatory cells in various conditions of urinary disorders. For example, elevated expressions of IL-17A, IL-17 F and IL-17RC and increased numbers of macrophages were observed in bladder cancer. One possible explanation may be that IL-17A and IL-17 F and IL-17RC can be expressed by these macrophages in bladder cancer. However, expression of IL-17A, IL-17RA and IL-17RC also increased in polyps but with the downgrade of numbers of macrophages. This suggests that macrophages might not be major source of these members of IL-17 family in polyps.

Additionally, other changed abnormal structural cells such as endothelial cells and fibroblasts were also observed in bladder cancer, which might contribute to the increases of immunoreactivity for IL-17A, IL-17 F and IL-17RC. It has been known that IL-17A and IL-17 F can bind to both IL-17RA and IL-17RC. For example, IL-17 F exerts effects on angiogenesis, while the elevated vessels in turn contribute to elevation of IL-17A or IL-17 F production in bladder cancer [27], which formats a positive feedback to promote malignant proliferation. In the present study, expression of IL-17A and IL-17 F increased but IL-17RA decreased in bladder cancer, suggesting that IL-17A and IL-17 F are possibly expressed by different profiles of cellular populations and mainly through binding IL-17RC to exert biological effects. Although we observed that some immunoreactivity for IL-17A and IL-17 F located in malignant cells, however, until far, there is lack of systemic study whether malignant cells in bladder cancer express IL-17A or IL-17 F. Clearly further experiments remain to be performed in this aspect.

IL-17E, through binding to its own receptor IL-17RB or IL-17RA, shows different properties from IL-17A and IL-17 F in tumor fields. Our data here showed that IL-17E and IL-17RB expression was elevated in cystitis but reduced in bladder cancer, possibly indicating that IL-17E might also be involved in the pathogenesis of benign urinary diseases. On one hand, IL-17E might participate in the pathogenesis of inflammation through acting on its receptor IL-17RB expressed on inflammatory cells and structural cells, which results in inflammation and alternation of structural cells. On the other hand, it is interesting that reduced expression of IL-17E and IL-17RB in bladder cancer. Such reduction in bladder cancer is possibly because the damaged or abnormal structural cells and malignant cells express less IL-17E and its receptor IL-17RB. This might affect anti-tumor effect of IL-17E in bladder cancer. Again, however, the details and underlying mechanisms of IL-17E in the pathogenesis of bladder cancer remain to be explored.

Our results also show that there were more vascular endothelial cells in bladder cancer compared with cystitis. It is well known that in bladder cancer, over-proliferation of cancer cells need more blood supplying, while these malignant cells and increased angiogenesis might affect other cell proliferation. On the other hand, vascular endothelial cells can express IL-17 family cytokine which might attract more inflammatory cells into malignant tissues and promote occurrence and development of bladder cancer. Apart of blood vessels, increased immunoreactivity for CD90^+^ fibroblasts was observed in bladder cancer, suggesting that these fibroblasts might also contribute to expression of IL-17 family ligands and receptors and possibly to pathogenesis of bladder cancer, through enhancing tumor growth [28, 29].

Since inflammatory microenvironment is a feature in urinary tract diseases while the relative members of IL-17 cytokine family closely link with inflammation, we also examined the status of common inflammatory cell types in the present study. The increased numbers of CD3^+^ T lymphocytes and CD68^+^ macrophage in bladder cancer suggest that these cells might participate in the process of bladder cancer. Macrophage is an important component of the inflammatory and tumor microenvironment and plays a key role in the progression of tumors. It has been shown that macrophage has dual function according to different tumor types [30]. Its function is, however, extremely complex and has not been elucidated till now. Neutrophil infiltration is a typical characteristic of acute inflammation. We also observed that there were more neutrophils in cystitis tissue than polyp and cancer, accompanied with increased IL-17RA, IL-17E and IL-17RB expression. Although neutrophils might not express IL-17E, these cells do express IL-17RA/or IL-17RB.

In addition, close correlations of expression of IL-17A-IL-17RA and IL-17E-IL-17RB suggest that different signaling of IL-17A-IL-17RA and IL-17E-IL-17RB might play an important role in bladder cancer. It is known that smooth muscle can express IL-17E. Thus, it is reasonably to presume that these cells might be a major cellular source of IL-17E, which may partly explain that the decreased IL-17E expression is a part of result due to the reduction of smooth muscle in bladder cancer. At the mean time, slight but significant correlation between immunoreactivity of IL-17 F and the numbers of CD3^+^ T lymphocytes was observed in bladder cancer, suggesting that CD3^+^ T lymphocytes might also be a major cellular source expressing IL-17 F. Whether these IL-17 F^+^ CD3^+^ T cells are a subgroup of Th17 cells remains to be investigated.

Obviously our study has some limitations. Firstly, it is still unknown for certain whether the specific index assessed by immunohistochemistry has been accurately identified. Clearly other experiments need to be done to confirm the roles of IL-17A and IL-17E signals in bladder cancer occurrence and development. Secondly, there were no entirely normal bladder specimens as controls, which might affect the comparison among the groups.

## Conclusion

Taking together our data indicate that changes of inflammatory and structural cells might be associated with the variable expression of IL-17 family cytokines, while increased blood endothelial cells and fibroblasts might be associated with bladder cancer occurrence and development.

## Additional file

Additional file 1: Table S1. Antibodies used in the present study. (DOC 16 kb)

## Abbreviations

BCG: Bacillus Calmette-Guéri
DAB: Diaminobenzidine
IFN: Interferon
IL-17: Interleukin-17
IL-17 F: Interleukin-17 F
IL-17 F: Interleukin-17 F
IL-17A: Interleukin-17 A
IL-17E: Interleukin-17 E
IL-17RA: Interleukin-17A receptor
IL-17RB: Interleukin-17 B receptor
IL-17RC: Interleukin-17C receptor
IL-17RE: Interleukin-17E receptor
IL-6: Interleukin-6
MC: Mast cell
NEU: Neutrophil
SM: Smooth muscle
TGF-β: Transforming growth factor β
TNF-α: Tumor necrosis factor-α
