# Supplementary material for: Immune analysis of expression of IL-17 relative ligands and their receptors in bladder cancer: comparison with polyp and cystitis
Source: BMC Immunol. 2016 Oct 3;17:36. doi: 10.1186/s12865-016-0174-8 (PMC5048669; doi:10.1186/s12865-016-0174-8)
Supplement: Additional file 1: Table S1. — Antibodies used in the present study. (DOC 16 kb) [file 12865_2016_174_MOESM1_ESM.doc]

# S-tab1 Antibodies used in the present study

| Antibody | Isotype | Item  number | Dilution | Sources |
| --- | --- | --- | --- | --- |
| Anti-IL-17A | Rabbit-IgG | NBP1-42746 | 1:100 | Novus Biologicals（USA） |
| Anti-IL-17E | Mouse-IgG1 | NB100-56541 | 1:8000 | Novus Biologicals（USA） |
| Anti-IL-17F | Mouse-IgG1 | NBP2-21684 | 1:800 | Novus Biologicals（USA） |
| Anti-IL-17RA | Goat-IgG | ab133416 | 1:60 | Abcam (Hong Kong) Ltd China） |
| Anti-IL-17RB | Mouse-IgG1 | NBP1-39952 | 1:200 | Novus Biologicals（USA） |
| Anti-IL-17RC | Rabbit-IgG | NBP1-83112 | 1:60 | Novus Biologicals（USA） |
| PECAM-1（Anti-CD31） | Mouse-IgG1 | SC-53411 | 1:25 | Santa Cruz Inc（USA） |
| Anti-CD90/Thy1 | Rabbit-IgG | NBP1-42068 | 1:200 | Novus Biologicals（USA） |
| Anti-alpha smooth muscle actin | Rabbit-IgG | ab32575 | 1:500 | Santa Cruz Inc（USA） |
| Anti-CD3 | Mouse-IgG2a | ab699 | 1:30 | Abcam (Hong Kong) Ltd（China） |
| Anti-CD68 | Mouse-IgG | sc-70761 | 1:800 | Santa Cruz Inc（USA） |
| Anti-Neutrophil Elastase | Rabbit-IgG | Ab21595 | 1:500 | Abcam (Hong Kong) Ltd（China） |
| Anti-Mast Cell Tryptase | Rabbit-IgG | Ab64192 | 1:2000 | Abcam Ltd（USA） |
